# Improving Cycling Behaviors of Dockless Bike-Sharing Users Based on an Extended Theory of Planned Behavior and Credit-Based Supervision Policies in China

**DOI:** 10.3389/fpsyg.2019.02189

**Published:** 2019-10-01

**Authors:** Lanying Sun, Xing Zhou, Zhaohui Sun

**Affiliations:** ^1^School of Marxism, Tianjin University, Tianjin, China; ^2^College of Management and Economics, Tianjin University, Tianjin, China

**Keywords:** dockless bike-sharing, social-psychological factors, users’ civilized cycling intention and behavior, effectiveness of credit-based supervision policies, sharing organization

## Abstract

Motivating users’ civilized cycling plays a significant role in alleviating the troubles of dockless bike-sharing programs (DBSPs) and promoting the sustainable development of bike-sharing organizations. Based on the theory of planned behavior (TPB) and observed practices in China, this study develops a theoretical framework to examine how attitudes (ATT), subjective norms (SN), perceived behavioral control (PBC), and personal norms (PN) motivate users’ civilized cycling behavior through civilized cycling intentions. Furthermore, the moderating effect of perceived policy effectiveness (PPE) between users’ civilized cycling intention and their actual behavior is tested. Using structural equation model-multiple group analysis (SEM-MGA) for a sample of 874 valid questionnaire responses in Beijing and Shanghai, China, our results reveal that (1) ATT, PBC, and PN are positively related to both users’ civilized cycling intentions and their actual behavior, while SN positively affect users’ civilized cycling intention only; (2) users’ civilized cycling intentions mediate the relationship between the four influencing factors and their actual behavior; and (3) PPE plays a moderating role for the effect of users’ civilized cycling intentions on their actual civilized cycling behavior. Our results indicate that the four influencing factors can encourage users’ civilized cycling behavior, especially when civilized cycling intention exists. Policies like credit-based supervision mechanisms could promote users’ civilized-cycling intentions, which could then be transformed into actual behavior.

## Introduction

As the trend of urbanization continues, issues such as traffic congestion, air pollution, and energy consumption seriously threaten the sustainable development of our cities. Bicycle-sharing systems play a significant role in urban transportation systems because they can reduce the dependence on cars and avoid the “great urban disease” (Fishman et al., 2014; Buehler et al., 2016; Federico et al., 2016). Since the first bike-sharing program, called the White Bicycle Plan, began operating in Amsterdam in 1965, bike-sharing has gained worldwide popularity. In 2014, about 855 cities throughout the world operated such programs, with a total of 946,000 bikes in use (Fishman, 2016). But traditional bicycle-sharing is constrained by the capacity of stations, limiting potential improvements for short distance travel. In the past 4 years, a new generation of dockless bicycle-sharing programs (DBSPs), such as Mobike and Ofo, has emerged and has experienced a period of rapid expansion, leading to a revival of the humble bike across China (Si et al., 2019). According to the report of iiMedia Research, the scale of Chinese shared bikes users has reached 0.235 billion people in 2018, and it would increase to 0.259 billion in 2019 (iiMedia Research, 2018). Currently, bike-sharing schemes cover more than 300 cities in China and have expanded overseas to 21 countries, such as Singapore, the United States, the United Kingdom, etc. (State Information Center, 2019). In the United States, for example, at least 57 cities now operate DBSPs (CBS News, 2018). Compared with traditional public bike-sharing, DBSPs allow people to unlock GPS-enabled bikes with their smartphones, and drop them off almost anywhere without the need to park at a dock. This huge advantage efficiently solves the “last mile” problem, which refers to the final leg of urban dweller’s journey (Li et al., 2018). What is more, the new generation of DBSPs encourages travelers to choose low-carbon travel modes by offering many personal benefits, including cost savings, flexibility of travel, and a healthy and low-carbon environmental lifestyle (Nikitas, 2018; WRI and Mobike, 2018).

Nevertheless, the rapid development of bicycle-sharing schemes has also created problems such as illegal parking, traffic violations, vandalism, and theft (Jia et al., 2018; Ma et al., 2018). For example, individuals have damaged some bikes in China by defacing the QR code or by attaching their own locks to the bikes to ensure their personal use. Many subway stations have become crowded with sharing bikes, degrading the urban esthetic environment and blocking pedestrian traffic. Similar situations have happened in other countries. According to CBS News (2018), dockless sharing bicycles in some cities in the United States (e.g., San Francisco) have earned the nickname “litter bikes” because of users’ random parking. Some people put the bikes in the middle of the street, in their backyards, or even up a tree. In other words, riders’ uncivilized behaviors threaten the safety and welfare of the public. Considering these negative externalities, some cities have decided to ban dockless shared bicycles, as Amsterdam did in September 2017 (Van Roy, 2017). In China, the Ministry of Transport (2017) and ten other departments jointly announced guidance for “encouraging and standardizing the development of bicycle-sharing” in August 2017.^1^ Many local governments (Beijing, Shanghai, etc.) are taking corresponding measures to force operators to strengthen the management of the bicycle-sharing systems through limits on the number of releasing bikes, real-name registration system for users, etc. Bicycle-sharing companies like Ofo and Mobike have also begun to apply electronic fence technology and to set up urban recommended parking stations to guide users to ride civilly (Yao et al., 2019). Unfortunately, illegal parking, traffic violations, vandalism, and theft still occur frequently (Shen et al., 2018). Since 2017, traffic management departments and bike-sharing companies like Mobike have started practicing cooperative management and have taken a series of credit-centered supervision measures to guide users’ cycling. Thus, exploring factors influencing users’ civilized cycling behavior and examining the effectiveness of existing supervision policies are critical steps to achieving dockless bike-sharing sustainability (Jia et al., 2018; Yang and Zhu, 2018; Yao et al., 2019).

While previous research has largely focused on traditional bike-sharing schemes (Fishman, 2016; Mátrai and Tóth, 2016; Faghih-Imani et al., 2017; Bettina et al., 2019), DBSPs are quite different. Since 2016, more studies on new-generation of DBSPs have been reported with bike-sharing’s widespread expansion. Several studies investigate the barriers, facilitators and determinants to the sustainable development of DBSPs (Nikitas, 2018; Shen et al., 2018; Shi et al., 2018). Scholars such as Shen et al. (2018) have found that bike fleet size, surrounding built environment, and supportive cycling facilities improve the usage of DBSPs. Conversely, weather conditions, such as rainfall and high temperatures, have a negative influence on them. Shi et al. (2018) identified stakeholder-associated factors in achieving DBSP sustainability and further classified them into six challenges (e.g., quantity control, commuting preferences of residents, lack of ancillary infrastructure, and parking management, etc.). The above-mentioned problems of bike-sharing are mostly caused by users’ uncivilized cycling intentions and behaviors. However, most behavioral studies have focused on users’ adopting behavior and choice intentions for DBSPs (Li et al., 2018; Cai et al., 2019). Very little research approaches users’ uncivilized cycling behaviors from a psychological perspective or examines the regulatory measures to control uncivilized behaviors (Chen et al., 2018; Jia et al., 2018). Therefore, it is important and necessary to examine the critical social-psychological factors influencing civilized use of sharing bikes and to explore the mechanism through which this behavior is achieved efficiently.

In this paper, an extended theory of planned behavior (TPB) and observed Chinese practices are applied to develop a conceptual model. We use the model to explore how these critical influencing factors improve users’ civilized cycling behavior through their intentions. More importantly, we further extend the model by adding perceived policy effectiveness (PPE) as a variable that moderates the relationship between users’ civilized cycling intentions and their actual behaviors (Wan et al., 2014; Xu et al., 2017). To achieve these research goals, we employ the structural equation modeling (SEM) and multiple-group analysis (Wang C. et al., 2018). From our results, we provide recommendations for policymakers and enterprise managers to solve the dilemma of negative externalities of sharing bikes.

The paper is organized as follows. See section “Theoretical framework and hypotheses development” lays out the theoretical framework and presents our hypotheses. An extended TPB model is used to carry out this research. See section “Materials and methods” describes the data collection, questionnaire development, and the methodology. See section “Results” presents the research results. Then, critical social-psychological factors as well as their relationships are discussed in see section “Discussion”. The final section summarizes the article, providing corresponding management strategies for promoting the development of DBSPs and summarizing the study limitations.

## Theoretical Framework and Hypotheses Development

### Theory of Planned Behavior

Theory of planned behavior is a theory of social psychology that focuses on the determinants of an individual’s behavior (Ajzen, 1991). This theory was developed by Ajzen (1991) as an extension of the theory of reasoned action. The TPB affirms that intention (IN) is the most direct and important precondition for people to generate specific behaviors (BE). IN can be predicted by the attitude toward the behaviors (ATT), subjective norms (SN), and perceived behavioral control (PBC). In general, TPB predicts that a more positive attitude, stronger SN, and greater PBC will boost one’s intention of performing a given behavior (Ajzen, 2011). Over the past decades, The TPB has been widely used in various research fields such as traffic, purchasing, and environmental protection etc. (e.g., Xu et al., 2017; Du et al., 2018; Cai et al., 2019). Many studies have utilized TPB to explain pro-cycling behaviors (Bruijn et al., 2009; Acheampong, 2017; Katharina, 2018). Therefore, TPB is perfectly applicable to users’ cycling behaviors.

### Hypotheses Development

#### Users’ Civilized Cycling Behaviors

Due to the great benefits of civilized cycling to traffic environmental sustainability (e.g., ease traffic congestion), users’ civilized cycling behavior is considered as one aspect of pro-environmental behavior which refers to activities “that consciously seek to minimize the negative impact of one’s actions on the natural and built world” (Kollmuss and Agyeman, 2002). In this study, Users’ civilized cycling behaviors are defined as those behaviors that voluntary compliance with relevant cycling regulation and social norms for the sake of public morality (Jia et al., 2018). Specifically, it refers to users’ responsible cycling behaviors, such as parking bikes as required, abiding by traffic rules, taking care of the sharing bikes, and not engaging in theft or vandalism. We first gained the users’ misbehaviors or misconduct behaviors based on related literature and interviews with experts on cycling research (Li et al., 2018; Ma et al., 2018). Second, we further classified and merged these behaviors. At last, the principal users’ behaviors that affect the civilized cycling were determined (e.g., Wang Y. et al., 2018).

#### Attitudes Toward Users’ Civilized Cycling Behaviors

Attitudes refer to an individual’s positive or negative evaluation of implementing a certain behavior under consideration (Ajzen, 1991). In relation to civilized cycling by users of sharing bikes, attitudes represent users’ comprehensive subjective evaluations (e.g., good/bad, convenient/inconvenient, likable/disgusting, supportive/opposed, etc.). TPB suggests that people’s attitudes toward specific behaviors (e.g., civilized cycling) will drive their intentions to perform those behaviors, which have been verified by related research in the case of cycling behavior (Acheampong, 2017; Yang and Zhu, 2018). Attitude includes both affective and instrumental aspects (French et al., 2005). Affective attitude refers to an individual’s feelings or emotions (e.g., good, fun, or supportive) toward a certain object. Cognitive consideration of the extent to which performing the behavior is beneficial or disadvantageous also exists in a person’s behavioral beliefs. Therefore, more favorable attitudes toward a behavior increase the likelihood that an individual will commit it (Armitage and Conner, 2001). This conclusion is confirmed by many relevant studies. For example, road users’ attitudes will have an impact on their actual behaviors (Boudrifa et al., 2012; Katharina, 2018; Shi et al., 2018). Based on the discussion above, the following hypotheses are proposed:

**Hypothesis 1a**: Users’ attitudes toward civilized cycling of sharing bike will have a positive direct effect on their civilized cycling intention.

**Hypothesis 2a**: Users’ attitudes toward civilized cycling of sharing bike will have a positive direct effect on their civilized cycling behavior.

#### Subjective Norms

Subjective norms represent a kind of pressure from others or social groups (Ajzen, 1991). People are most likely to perform certain behaviors in accordance with suggestions of important individuals or perceived social pressure (Ajzen, 1991). For example, if civilized cycling is generally perceived by a group of people as consisting of “idiotic deeds” which may inconvenience some individuals, then the common belief could discourage people from using sharing bikes civilly. Since the beginning of shared bike use in China, extensive publicity and education activities of civilized cycling have been carried out by Chinese government and news media to solve the dilemma of negative externalities of sharing bikes. Currently, a relatively friendly cycling atmosphere has been achieved in the whole society, which influences and regulates users’ cycling behaviors by functioning as informal social controls. Also, related studies have verified that SNs impact the civilized cycling intentions and their actual behaviors of sharing bike users (Federico et al., 2016; Jia et al., 2018; Yang and Zhu, 2018). Thus, based on TPB, we propose the following hypotheses.

**Hypothesis 1b**: Users’ SN will have a positive direct effect on their civilized cycling intention.

**Hypothesis 2b**: Users’ SN will have a positive direct effect on their civilized cycling behavior.

#### Perceived Behavioral Control

Perceived behavioral control refers to the degree of ease or difficulty perceived by a person when performing a certain behavior (Ajzen, 1991). PBC includes control beliefs and perceived strength. Control beliefs refer to the factors that are needed to conduct a certain behavior. Perceived strength is defined as individuals’ estimates of their abilities to control these factors to implement certain behavior in current conditions. Extensive research has shown that a high-level of PBC leads to stronger behavioral intention and behavior performance (Ajzen, 1991; Shi et al., 2018; Wu et al., 2019). In this study, if the user is confident enough and has strong capacities to ride sharing bikes civilly and the city provides sufficient cycling conditions (e.g., proper parking spaces), civilized cycling behaviors can be generated easily. Thus, the following hypothesizes are proposed:

**Hypothesis 1c**: Users’ PBC will have a positive direct effect on their civilized cycling intention.

**Hypothesis 2c:** Users’ PBC will have a positive direct effect on their civilized cycling behavior.

#### Personal Norms

Personal norms (PN) is individual’s internal moral duty and responsibility to conduct or refrain a certain behavior (Ajzen, 1991). Compared with SN, PN is often trigged by people’s own obligation rather than external pressures. Previous literatures have revealed that PN is a vital factor to influence the pro-cycling behaviors (Bamberg et al., 2007; Kim et al., 2017). Thøgersen (2006) also found that PN is more powerful to predict actual environmentally responsible behaviors. In China, bike sharing is often called “a mirror reflecting national quality” which emphasizes the important influence of personal moral quality on civilized cycling of sharing bikes. Users who have strong personal norm may own strong moral obligations to ride bikes civilly. Instead, users who have weak personal norm will impair them to perform civilized cycling. This is because uncivilized cycling of sharing bikes may lead riders to feel guilty and shame as such behaviors against their internal moral duty. Hence, it is reasonable to predict that PN will positively affect both users’ civilized cycling intentions and their actual behaviors. According the discussion above, this hypothesis is proposed:

**Hypothesis 1d**: Users’ PN will have a positive direct effect on their civilized cycling intention.

**Hypothesis 2d**: Users’ PN will have a positive direct effect on their civilized cycling behavior.

#### The Mediating Effect of Users’ Civilized Cycling Intention

If a variable is essential for an independent variable to influence a dependent variable, then this variable is known as mediator (Qu et al., 2014). Intention is an individual’s perceived subjective motivation for engaging in a specific behavior (Ajzen, 1991, 2011). According to the TPB model, the intention (IN) is the most direct precondition for people to generate actual behavior (BE), which can be predicted by attitude, social norms, and PBC. In other words, IN exerts a prominent mediating effect on the relationship between ATT, SN, PBC, and BE (Ajzen, 1991; Qu et al., 2014; Wang C. et al., 2018). Users’ civilized cycling behavioral intention reflects their willingness to spend time, money, and effort in the civilized use of sharing bikes. Extant research reports that users’ civilized cycling intention is the best predictor of their behavior, and their intention is affected by both internal and external factors (Jia et al., 2018; Cai et al., 2019). Specifically, the civilized cycling intention efficiently transforms a cyclist’s attitude, PN, etc., into actual civilized cycling behavior. Therefore, we propose the following hypotheses.

**Hypothesis 3**: Users’ civilized cycling intention will have a positive direct effect on their actual behaviors.

**Hypothesis 4a**:Users’ civilized cycling intention mediates the relationship between attitudes toward civilized cycling and their actual behaviors.

**Hypothesis 4b**:Users’ civilized cycling intention mediates the relationship between SN and their actual behaviors.

**Hypothesis 4c**:Users’ civilized cycling intention mediates the relationship between PBC and their actual behaviors.

**Hypothesis 4d**:Users’ civilized cycling intention mediates the relationship between PN and their actual behaviors.

#### The Moderating Effect of Perceived Policy Effectiveness

A moderating effect means that a variable as a moderator can strengthen or weaken the relation between an independent variable and a dependent variable (Qu et al., 2014). Sharing bike users’ cycling behavior is not completely independent but is affected by contextual factors like policies, culture, etc. (Yao et al., 2019). For example, Wan et al. (2014) demonstrated that PPE have a positive influence for environmental decision-making. Besides, significant evidence has shown that policies and regulations can be highly successful in promoting the transformation of intention to behaviors in many areas (e.g., Wan and Shen, 2013; Xu et al., 2017). So, we employ the PPE as moderator in this study.

Since August 2017, the Chinese Ministry of Transport has issued policy documents providing “the guidance about encouraging and standardizing the development of bicycle-sharing.” Afterward, parts of local traffic management departments and bike-sharing companies, like Mobike, cooperated together to take a series of supervision measures to correct users’ uncivilized cycling behaviors. The core of these measures is a new system of credits to reward appropriate behaviors and to punish violations. In September 2017, the State Information Center signed a credit information sharing agreement with Mobike, Ofo, Hellobike, and ten other major bicycle enterprises to establish a credit-based supervision mechanism^2^. Therefore, assessing the effectiveness of these credit-based supervision policies will be important for promoting the establishment and business application of a national personal credit system.

In this study, PPE is defined as a user’s favorable or unfavorable evaluation of the effects of these credit-based supervision policies (Wan et al., 2014; Xu et al., 2017). PPE may not only function as a direct predictor of behavior (Du et al., 2018) but also moderates the influence of other variables on their behaviors. If users perceive a stronger and more effective motivation for these credit-based supervision measures, the intention to perform civilized cycling behaviors will be enhanced and the intention-behavior gap will be weakened. Based on the discussion above and previous research frameworks (e.g., Wang C. et al., 2018), we posit the following hypothesis.

**Hypothesis 5:** PPE will positively moderate the relationship between users’ civilized cycling intention and their actual behaviors.

#### Control Variables

Control variables have the potential to confound the effects of determinants on the explanatory variable (Mehta, 2015). Currently, numerous studies related to cycling behaviors have demonstrated that demographic variables have an important impact on an individual’s behavioral intention and actual behaviors (Holland and Hill, 2007; Bernhoft and Carstensen, 2008; Cordellieri et al., 2016; Wu et al., 2019). For example, Bernhoft and Carstensen (2008) found that younger people are more likely to adopt unsafe riding behaviors because they more often choose a fast passage rather than pavement. Jia et al. (2018) found that cyclists with higher levels of education have better quality and control ability to use sharing bikes civilly. Also, studies have revealed that males are more prone to ride while using cell phones than females, and high-income people pay more attention to cycling safety than low-income people do (Holland and Hill, 2007; Wu et al., 2019). Therefore, we assume that gender, age, educational level, and income impact civilized cycling intention and behavior (e.g., Acheampong, 2017; Katharina, 2018).

### An Integrated Theoretical Framework

**FIGURE 1:**
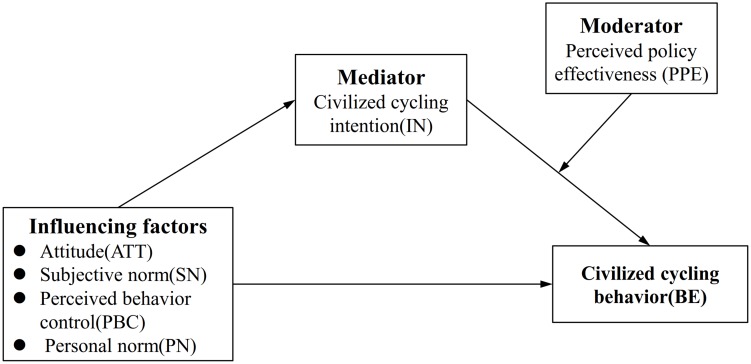
A theoretical framework of users’ civilized cycling behaviors.

Previous literature has demonstrated that the TPB model is suitable for predicting cycling behaviors (Yang and Zhu, 2018; Cai et al., 2019). However, this model needs to be extended to improve its predictive ability. In this study, an integrated theoretical framework is adopted that considers socio-demographic factors (attitude, SN, PBC, and PN), situational elements (e.g., policy), and demographic variables, as shown in Figure 1. This framework can be used in a wide range of situations and can better predict cycling behavior.

## Materials and Methods

### Participants and Procedure

To test the theoretical framework, a survey was conducted in Beijing and Shanghai, which are two of the four municipalities of China. These two cities were the birthplace of Ofo and Mobike. Beijing had more than 2.35 million sharing bikes in circulation at the peak in September 2017, which severely exceeded the capacity of city streets and sidewalks. Currently, about ten bicycle-sharing companies, including Ofo, Mobike and Hellobike, operate roughly 1.91 million shared bicycles in Beijing (Beijing Municipal Commission of Transport, 2018). Shanghai had 1.78 million shared bicycles at its peak level, but now the number has dropped to 1.15 million (Shanghai Municipal Commission of Transport, 2017). While Beijing and Shanghai citizens have enjoyed the convenience of bicycle-sharing systems, they have also been negatively affected by uncivilized cycling.

A 28-item questionnaire was designed to collect data from a sample of residents of Beijing and Shanghai. Before the formal investigation, we conducted random one-on-one interviews with written informed consent, obtaining 30 questionnaires in the pre-survey. After conducting a content analysis of the pilot questionnaires and consulting with survey experts, certain items were further modified within the questionnaire. It should be noted that, in the first page of the questionnaire, we introduced our research purpose and plans to each of the participants and then secured their written informed consent to participate in this academic research. Non-adult participants were allowed to answer this questionnaire only after getting their parent or guardian’s written informed consent. According to the criteria of the research group, only the responses where consent was granted could be used for data analysis. So, we confirmed that written informed consent was obtained from all adult participants and parents or guardians of all non-adult participants. The internal Ethics Committee of Tianjin University approved this study including all consent procedures.

The two wave data collecting method was utilized to avoid bias of the results from self-reported data (e.g., Xiao et al., 2017). The survey was initially conducted from October to December of 2018 and second data collection was conducted during August 2019. Our survey consisted of online random sampling and the questionnaire on site. We collected most of our data on weekend afternoons due to citizens’ greater willingness to participate in a survey in their spare time. For the specific survey site, we divided the Beijing study region into eight urban districts (Dongcheng, Xicheng, Chaoyang, Haidian, Chongwen, Xuanwu, Fengtai, and Shijingshan) and 32 collection blocks. Similarly, Shanghai was divided into seven urban districts (Huangpu, Jing’an, Xuhui, Changning, Yangpu, Hongkou, and Putuo) and 28 collection blocks. On this basis, thirty collection blocks including 16 blocks in Beijing and 14 blocks in Shanghai were chosen at random. To ensure data accuracy, we sent 20 graduate students who had passed our training to the 30 collection blocks. Each group of two graduate students was responsible for street-intercept interviews in their collection block. A total of 1020 questionnaires were obtained, yielding 874 valid questionnaires, with an effective response rate of 85.7% (see Table 1). The demographic characteristics of the sample, including gender, age, education, and income, were recorded. Of the respondents, 47.71% (*n* = 417) were male and the others female (*n* = 457). The age grouping with the greatest number of responses was 19–25 years of age (38.44%), followed by 26–35 years of age (28.26%), and the average age was about 25 years. As for education and income, almost half of the participants hold an undergraduate degree (43.94%) and 28.26% were graduate students. Further, the largest response group reported a monthly income below CNY3000 (37.19%), and the next group represented 24.94% and reported an income above CNY9000. The middle-income group (monthly income 3001–6000 yuan or 6001–9000 yuan) accounts for 37.87%.

**TABLE 1:** Sample demographics (*N* = 874).

**Background**	**Category**	**Frequency**	**Percentage (%)**
Gender	Male	417	47.71
	Female	457	52.29
Age	12–18	157	17.96
	19–25	336	38.44
	26–35	247	28.26
	36–45	93	10.64
	>46	41	4.69
Education	High school or below	167	19.11
	Junior college	76	8.70
	College	384	43.94
	Master or above	247	28.26
Income	<=3000	325	37.19
	3001–6000	148	16.93
	6001–9000	183	20.94
	>9000	218	24.94

### Instrument and Measure

Drawing on previous studies and the particular case of this research, we construct our questionnaire items based on the principles of target, action, context, and time (TACT) (Ajzen, 2006). For example, we could ask individuals their attitudes toward civilized cycling behavior when riding shared bikes in the past 4 years. In this instance, the target (participant), action (civilized cycling), context (when riding shared bikes), and time (in the past 4 years) are clearly defined. However, the details in these statements are too complicated to confuse respondents, which may lead to neglecting the focus of attention. Considering the fact that the context and time components remain the same for each item, we outline them at the beginning of every statement sections of the questionnaire. The target and the action are defined individually for each item (e.g., Williams et al., 2012). At the same time, the foreign scales are translated into Chinese using the standard translation-back-translation procedure to eliminate the cultural differences between the East and the West (Brislin, 1980).

Following the above procedures, we design the measurement scales including 28 questions for the chosen seven constructs. The relevant items and their sources are shown in Table 2. The measures of attitudes, SN, PBC, PN, and PPE are assessed using a 7-point Likert-type scale ranging from “Strongly disagree” (1) to “Strongly agree” (7). Unlike the measures of social-psychological factors, users’ civilized cycling behavior is assessed using a different 7-point Likert-type scale ranging from “never” (1) to “usually” (7). Similarly, users’ civilized cycling intentions are assessed with a 7-item scale ranging from 1 (very reluctant to) to 7 (very glad to), with 4 serving as neutral. It should be noted that four items (BE1, BE2, BE3, and BE4) are reverse scored to evaluate levels of a user’s civilized cycling behavior.

**TABLE 2:** Constructs and scale items.

**Dimensions and items**	**References**
**Attitude toward civilized cycling (ATT):**	
1. Civilized cycling is a good thing	Ajzen, 1991; Deng et al., 2016
2. Civilized cycling is worthwhile to provide a convenient traffic environment	
3. Civilized cycling makes me happy	
4. I support civilized cycling behaviors	
**Subjective norms (SN):**	
1. My family members greatly influence my choice to engage in civilized cycling behaviors	Ajzen, 1991; Deng et al., 2016
2. Colleagues and friends greatly influence my choice to engage in civilized cycling behaviors	
3. The news media greatly influences my choice to engage in civilized cycling behaviors.	
4. Government greatly influences my choice to engage in civilized cycling behaviors.	
**Perceived behavioral control (PBC):**	
1. It is easy for me to engage in civilized cycling behaviors	Ajzen, 1991;
2. If I want to, I could easily engage in civilized cycling behaviors.	Renger and Reese, 2017
3. It is mostly up to me whether I engage in civilized cycling behaviors.	
4. Urban cycling facilities such as cycle lanes, public parking areas, etc., can meet the needs of my daily cycling completely.	
**Personal norms (PN):**	
1. I feel an obligation to choose a suitable parking area instead of random parking.	Nordlund and Garvill, 2003
2. Regardless of what other people do, because of my own values/principles, I feel that I should not steal sharing bicycles.	
3. I feel that it is important to cherish sharing bicycles in the process of riding to ensure its normal use.	
4. Civilized cycling is in accordance with the value of environmental protection	
**Perceived policy effectiveness (PBC):**	
1. The user’s credit score system is necessary in order to encourage people to choose civilized cycling.	Wan et al., 2014; Xu et al., 2017
2. The user’s credit score system is valid in order to encourage people to choose civilized cycling.	
3. The government’s promotion of user’s credit score system helps citizens understand the importance of civilized cycling.	
4. The punitive damages of user’s credit score system can control my illegal parking behaviors.	
**Civilized cycling intention (IN):**	
1. I am willing to cherish the sharing bikes in the process of riding.	Ajzen, 1991; Wang Y. et al., 2018
2. I am willing to spend time to park the sharing bikes in a suitable parking area.	
3. I am willing to stop others from vandalizing and stealing the sharing bikes.	
4. I am willing to participate in a related survey to offer some advice for guiding others in civilized cycling.	
**Civilized cycling behavior (BE):**	
1. Have you parked sharing bicycle illegally?	Ajzen, 1991; Wang Y. et al., 2018
2. Have you damaged sharing bicycle such as scratching off the QR code, locking bikes with own chain lock, etc.?	
3. Have you hidden the sharing bicycle in or near your own home to facilitate yourself?	
4. Have you broken the sharing bike-using policy, including violating the traffic rules, or riding out of the bicycle-sharing service area?	

### Analytical Method

We used the SPSS 21 and AMOS 21 software in our data analysis. To achieve the study objectives, structural equation models (SEM) are used to test the research hypotheses. The reliability and validity are initially evaluated by confirmatory factor analysis (CFA). After that, we assess the proposed model and analyze the direct effect of influencing factors on civilized cycling intention and actual behaviors. Thirdly, powerful bootstrapping methodology is adopted to test the statistical significance of indirect effects in mediation models. Finally, the moderating impact of PPE between users’ civilized cycling intention and their actual behaviors is examined using multiple-group analysis (MGA) with AMOS.

## Results

### Reliability and Validity Testing

The study constructs are assessed for composite reliability (CR), convergent validity, and discriminant validity. A summary of the findings from the CFA is displayed in Table 3. Findings indicate that all composite reliability values are all within the range of 0.894 to 0.933, which are greater than the minimum threshold of 0.60. Factor loadings for the items are between 0.776 and 0.910 and exceed the critical value of 0.60. All standardized loadings are significant (*p* < 0.001). Next, the average variance extracted (AVE) values are also calculated. All AVE values for latent variables, which range from 0.679 to 0.777, are above the suggested cut-off of 0.50 (Fornell and Larcker, 1981). The square root of each variable’s AVE is further compared with the correlations between variables. If the square root of a variable’s AVE is higher than correlation coefficients involving that variable, it indicates convergent and discriminant validity exists (Fornell and Larcker, 1981). For example, the square root of the AVE of SN (0.858) is higher than the correlation coefficients involving it (0.354; 0.275; 0.273; 0.228; 0.354; and 0.281); so, the discriminant validity of SN is acceptable. Consequently, in this study, all measurements of the latent variables are demonstrated to have good reliability and validity.

**TABLE 3:** Confirmatory factor analysis for the survey instrument validity and reliability.

**Items**	**Std. FL**	**CR**	**AVE**	**1**	**2**	**3**	**4**	**5**	**6**	**7**
ATT	0.844–0.869	0.918	0.736	0.858						
SN	0.801–0.892	0.918	0.736	0.354^∗∗^	0.858					
PBC	0.856–0.910	0.933	0.777	0.480^∗∗^	0.275^∗∗^	0.881				
PN	0.776–0.870	0.894	0.679	0.395^∗∗^	0.273^∗∗^	0.393^∗∗^	0.824			
PPE	0.865–0.875	0.926	0.756	0.227^∗∗^	0.228^∗∗^	0.227^∗∗^	0.175^∗∗^	0.869		
IN	0.801–0.851	0.897	0.686	0.508^∗∗^	0.354^∗∗^	0.430^∗∗^	0.454^∗∗^	0.205^∗∗^	0.828	
BE	0.808–0.856	0.905	0.705	0.505^∗∗^	0.281^∗∗^	0.482^∗∗^	0.463^∗∗^	0.349^∗∗^	0.547^∗∗^	0.840
MEAN				5.458	5.165	5.559	5.526	6.078	5.929	5.293
SD				1.333	1.358	1.367	1.289	1.153	1.103	1.364

*(1) The items on the diagonal represent the square roots of the AVE; off-diagonal elements are the correlation estimates; SD, standard deviation; (2)ATT, attitudes; SN, subjective norms; PBC, perceived behavioral control; PN, personal norms; PPE, perceived policy effectiveness; IN, users’ civilized cycling intention; BE, users’ civilized cycling behavior; (3) ^∗^p < 0.05, ^∗∗^p < 0.01, ^∗∗∗^p < 0.001.*

### Model Analysis

The goodness of fit indices for the model are as follows: χ2 = 716.086; df = 309; *p* < 0.001; χ2/df = 2.317; GFI = 0.941; TLI = 0.969; CFI = 0.975; SRMR = 0.024; RMSEA = 0.039. The results are in accordance with the guideline of χ2/df < 5.00 (Wheaton et al., 1977). In addition, the GFI, TLI, and CFI are higher than the recommended value of 0.90 (Hooper et al., 2008; de Leeuw et al., 2015) The SRMR and RMSEA are below 0.08 (Hooper et al., 2008). The fit indices meet the recommended levels. Thus, the model is suitable to the analysis of the civilized cycling behavior of dockless bike-sharing users.

**FIGURE 2:**
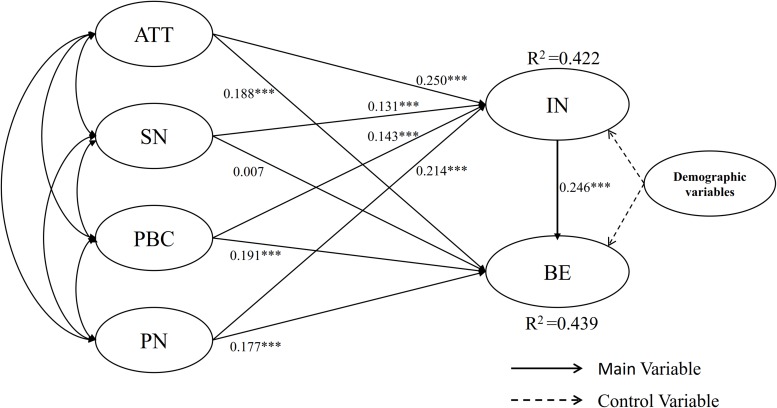
Results of the structural equation model. (1) ATT, attitudes; SN, subjective norms; PBC, perceived behavioral control; PN, personal norms; PPE, perceived policy effectiveness; IN, users’ civilized cycling intention; BE, users’ civilized cycling behavior; (2) ^∗^*p* < 0.05, ^∗∗^*p* < 0.01, ^∗∗∗^*p* < 0.001.

The hypothesized relationships in the proposed model are also tested. Figure 2 summarizes these results. All influencing factors including ATT (β = 0.250, *p* < 0.001), SN (β = 0.131, *p* < 0.001), PBC (β = 0.143, *p* < 0.001) and PN (β = 0.214, *p* < 0.001) are found to be significantly and positively associated with IN. The four influencing factors jointly explain 42.2% of the total variance of IN. That is, ATT, SN, PBC, and PN are significant predictors of IN. Hence, Hypotheses 1a, 1b, 1c, and 1d are supported. Moreover, ATT (β = 0.188, *p* < 0.001), PBC (β = 0.191, *p* < 0.001), and PN (β = 0.177, *p* < 0.001) each have significant positive effects on BE. Hypotheses 2a, 2c, and 2d are supported, However, SN (β = 0.007, *p* = 0.823) does not exert a statistically significant influence on BE. Thus, Hypotheses 2b is not supported. The four influencing factors (ATT, SN, PBC, and PN) and IN together account for about 43.9% of the total variance in BE which is at an acceptable range. IN is positively related to BE (β = 0.246, *p* < 0.001), as suggested by Ajzen (2011); and Hypotheses H3 is supported.

### Mediating Role Test of Civilized Cycling Intention

Next, we analyze the mediation effect of IN in the extended TPB model and present the results in Table 4. We examine whether users’ civilized cycling intentions are essential for the transformation of influencing factors into civilized cycling behaviors. Among several methods of testing the mediation effect, the causal steps approach and the Sobel test have been used widely but have also been criticized by researchers (MacKinnon et al., 2002; Hayes, 2009). So, we adopt a bootstrapping method that results in more accurate confidence intervals for indirect effects (MacKinnon et al., 2004). We use AMOS 21 to calculate 5000 bootstrap samples. If zero is not between the lower and upper bound of the bias-corrected percentile and percentile, the mediation effect is shown to be significant at a confidence interval of 95%. As presented in Table 4, four factors including ATT, SN, PN, and PBC have indirect effects on BE through IN. Among them, the leading indirect decisive factor of BE is ATT, and its indirect effect is 0.064 (SE = 0.023, *Z* = 2.783). PN and PBC follow with indirect effects of 0.061 (SE = 0.019, *Z* = 3.211) and 0.034 (SE = 0.015, *Z* = 2.267), respectively. SN has the smallest effect on BE, as its indirect effect is 0.031 (SE = 0.013, *Z* = 2.385). For each of the four factors, the 95% confidence interval for the bias-corrected percentile and percentile does not contain zero. So, hypotheses 4a, 4b, 4c, and 4d are supported. However, the direct effect of SN on BE is 0.007 (SE = 0.038, *Z* = 0.184) and zero is contained within the lower and upper bound of the bias-corrected percentile and percentile, showing that SN has no significant direct impact on BE. In summary, the above results indicate that IN partially mediates the effects of AB, PBC, and PN on BE and fully mediates the relationship of SN and BE.

**TABLE 4:** Mediating effect results.

			**Product of coefficients**		**Bootstrapping**					
					**BC 95% CI**			**Percentile 95% CI**		
**Factor**	**Effect**	**Point estimate**	**SE**	**Z**	**Lower**	**Upper**	**P**	**Lower**	**Upper**	**P**
ATT	IE	0.064	0.023	2.783	0.028	0.121	0.000	0.024	0.113	0.001
	DE	0.197	0.068	2.897	0.067	0.329	0.006	0.063	0.324	0.007
SN	IE	0.031	0.013	2.385	0.012	0.065	0.001	0.010	0.059	0.002
	DE	0.007	0.038	0.184	–0.068	0.081	0.904	–0.064	0.085	0.821
PBC	IE	0.034	0.015	2.267	0.011	0.076	0.002	0.008	0.068	0.006
	DE	0.182	0.056	3.250	0.072	0.293	0.001	0.074	0.296	0.001
PN	IE	0.061	0.019	3.211	0.031	0.110	0.000	0.028	0.103	0.001
	DE	0.207	0.064	3.234	0.088	0.338	0.001	0.091	0.340	0.001

*(1) IE, indirect effects; DE, direct effects; S.E, standard error, BC, bias-corrected percentile; 5000 bootstrap samples. ATT, attitudes; SN, subjective norms; PBC, perceived behavioral control; PN, personal norms; PPE, perceived policy effectiveness; IN, users’ civilized cycling intention; BE, users’ civilized cycling behavior. (2) ^∗^p < 0.05, ^∗∗^p < 0.01, ^∗∗∗^p < 0.001.*

### Multiple-Group Analysis—Moderating Effect Verification of PPE

Although we have found that attitudes, SN, PBC, and PN can induce intentioned behaviors, the intention-behavior gap still remains (Du et al., 2018; Si et al., 2019). Therefore, contextual factors such as PPE are needed to induce civilized cycling behaviors (Wan and Shen, 2013; Yao et al., 2019). To test the moderating effect of PPE between civilized cycling intention and behavior, multiple-group analysis (MGA) with AMOS 21 is performed (e.g., Tarhini et al., 2015; Wang C. et al., 2018). First, the sample is divided into two groups on the basis of the 27% approach (Ross and Weitzman, 1964). Specifically, the top 27% of the mean value of PPE belong to the high-group (*N* = 285) and the bottom 27% go with the low-group (*N* = 328). Table 5 shows the results of the goodness-of-fit tests which indicate the model are fit for both groups.

**TABLE 5:** Goodness-of-fit test.

**Goodness- of-fit index**	**χ2/df**	**CFI**	**IFI**	**NFI**	**TLI**	**PGFI**	**SRMR**	**RMSEA**
Low group	1.446	0.966	0.967	0.900	0.959	0.683	0.039	0.040
High group	2.283	0.951	0.952	0.917	0.940	0.653	0.035	0.063

This research primarily assesses whether the PPE can moderate the IN-BE path. Therefore, only the difference between the limited structural weight model and the limited measurement weight model is considered. The results are Δχ2 = 122.612, Δdf = 17, *p* < 0.001 indicating the two models have a significant difference.

Furthermore, the pairwise parameter comparisons using critical ratios for differences (CRD) are also conducted to determine if the IN-BE path is different between the low-level group and the high-level group. The absolute value of CRD of the IN-BE path between the two groups is 3.833 (above the threshold of 1.96), showing significance at the 0.05 level (Tarhini et al., 2015; Wang C. et al., 2018). As shown in Table 6, PPE moderates IN-BE path. The IN-BE path coefficient of the high-level group (0.373) is greater than that of the low-level group (0.016). Hence, hypothesis 5 is supported; PPE positively moderates the relationship between users’ civilized cycling intentions and their actual behavior, such that the effect of IN on BE is enhanced by PPE.

**TABLE 6:** Multi-group analysis across the low and high group of PPE.

**Path**	**PPE**			**C.R. (low-high group)**
	**Low**	**High**	**Difference**	
IN-BE	0.016	0.373^∗∗∗^	0.357^∗∗∗^	3.833

*(1)BE, civilized cycling behavior; IN, civilized cycling intention; PPE, perceived policy effectiveness. (2)^∗^*p* < 0.05, ^∗∗^*p* < 0.01, ^∗∗∗^*p* < 0.001.*

## Discussion

To predict sharing bike users’ civilized cycling behavior, this study uses an extended TPB model to examine how attitude, SN, PBC, and PN shape their actual intentional behaviors. After controlling for demographic variables including gender, age, education and income, our results show that attitude, PBC, and PN are positively related to both users’ civilized cycling intentions and their actual behavior. SN has a positive impact on users’ civilized cycling intentions only. In addition, users’ civilized cycling intentions mediate the relationship between the four influencing factors above and their actual behavior. More importantly, PPE plays a moderating role in the effect of users’ civilized cycling intentions on their actual civilized cycling behavior.

### Theoretical Implications

This study has several important implications for research on sharing bike users’ civilized cycling behavior. The relationships among users’ individual factors are identified in this study. In accordance with prior research regarding cycling behaviors, attitude, SN, PBC, and PN positively affect users’ civilized cycling intentions (Chen et al., 2018; Huemer, 2018; Yang and Zhu, 2018). However, few researchers have examined the effects of attitude, SN, and PN on actual behavior. In this study, we find that attitude along with PBC and PN are also positively related to user’s civilized cycling behavior (Boudrifa et al., 2012; Shi et al., 2018). Among these factors, PBC exerts greater influence when comparing with attitudes and PN (Acheampong, 2017). However, SN do not have a significant direct influence on users’ civilized cycling behavior in the Chinese context (Hagger et al., 2011; Yang and Zhu, 2018). This result can be explained as follows. First, the sustainable development of DBSPs mainly relies on higher personal moral quality. Cyclists get little influence from others in the use of riding sharing bikes (Yang and Zhu, 2018). Second, the relevant rules and regulations for sharing bikes are still relatively new and imperfect, which limits the pressure they exert on users’ cycling behaviors. Finally, when deciding whether to engage in civilized riding when parking in practice, the cyclist is constrained by a lack of ancillary infrastructure and appropriate parking areas (Sun et al., 2017).

Besides the direct effects of ATT, SN, PBC, and PN, we verify that users’ civilized cycling intentions meditate the relationship between these four influencing factors and their actual civilized cycling behavior. Our results provide encouraging empirical evidence for the application of Ajzen’s (1991, 2011) TPB model to cycling, demonstrating that social-psychological factors (e.g., ATT, SN, and PBC) shape users’ civilized cycling intentions, which further influences their actual behavior (Huemer, 2018; Jia et al., 2018). This finding underscores the wide adaptability of TPB model in cycling behavioral research (Huemer, 2018; Yang and Zhu, 2018; Cai et al., 2019). In agreement with previous research, our results also confirm that users’ civilized cycling intentions are the most direct precondition for generating actual civilized cycling behavior and play an indispensable mediating role in the process of achieving their actual behavior (Jia et al., 2018; Yang and Zhu, 2018). That is to say, only when a user is motivated to have civilized cycling intentions can his or her actual behavior be realized. Furthermore, as the four influencing factors that can positively motivate users’ civilized cycling intentions, ATT, SN, PBC, and PN can induce their actual civilized cycling behavior partially or fully through the mediator of the intention (Boudrifa et al., 2012). Specifically, IN partially mediates the effects of ATT, PBC and PN on BE and fully mediates the effects of SN on BE. In comparison with views of Ajzen (1991, 2011), we demonstrate that IN plays a partial rather than full mediating role in the relationship between ATT and BE (e.g., Qu et al., 2014). Higher levels of ATT, SN, PBC, or PN are associated with greater mediating effects of intention, which increases the probability that cyclists undertake civilized cycling.

Finally, this research contributes to closing the intention-behavior gap by including the moderator role of PPE, which enhances the prediction ability of the TPB model (Wang C. et al., 2018). Although previous studies have verified that a significant increase in intention can induce a moderate change in behavior, a significant gap between what one intends to do and what is actually performed always exists (Tom and Brunton, 2017). However, this intention-behavior gap can be narrowed by adding additional variables (Mullan et al., 2013). In this research, we analyze the effect of PPE on users’ civilized cycling behavior. Our results show it positively moderates the relationship between civilized cycling intention and their actual behavior (Wang C. et al., 2018; Yao et al., 2019). This is consistent with previous studies in that situational factors like PPE play a vital role in users’ decision-making (Schneider and Ingram, 1990; Wan et al., 2014). The positive moderation effect suggests that credit-based supervision policies are helpful in facilitating intended civilized cycling behaviors (Xu et al., 2017; Yao et al., 2019).

### Practical Implications

This study also reveals some important practical implications for enterprise managers and policymakers. It is vital to improve users’ civilized cycling intentions by changing individuals’ attitudes toward DBSPs, as well as creating a good social atmosphere for civilized cycling. Currently, considering the serious troubles of DBSPs, governments should implement stricter credit-based supervision policies and measures to regulate users’ cycling behaviors. Such policies include establishing a platform to publish traffic information (e.g., parking area, usage evaluation, and personal credit information) (Shi et al., 2018), cooperating with companies to refine the supervision mechanism (Guo, 2017), and upgrading urban transportation systems to create cycling-friendly traffic environments. In the long run, measures should focus on improving users’ civilized cycling intentions by cultivating a culture that values green travel and cherishes DBSPs. Massive campaigns about the serious consequences for uncivilized cycling should be launched by governments to improve people’s moral quality gradually and to increase the trust level of society as a whole. Moreover, some economic incentives, such as discount rates, can also be provided by operators to encourage civilized cycling behavior. In fact, we do agree that Chinese cyclists’ behavior in the use of DBSPs has generally improved in recent years with regulatory actions of local governments. However, collaborative governance of DBSPs among government, corporations, and the public remains an area in need of improvement, which may the trend in the future. We should share and exchange information among stakeholders and make joint efforts to supervise the DBSPs, which are key points in promoting credit-based supervision systems in the future (Guo, 2017; Ma et al., 2018).

### Limitations and Future Research

Despite these contributions, this study contains several limitations. First, the data obtained are self-reported by citizens, so the potential for social desirability bias among the responses must be acknowledged. In future research, multiple data sources will be needed to assess users’ cycling behavior and to reduce the potential problems of self-reporting. Second, we observed five influencing factors that are considered as the most influential stressors of cycling behaviors in previous studies. In future work, more factors, especially other cultural variables, should be considered to improve the explanatory power of the model. In-depth studies would be valuable to examine the motivation mechanism of the users’ civilized cycling behavior further. Third, although the moderator variable of PPE is shown to be helpful in promoting users’ civilized cycling behavior, further analysis is needed on how it influences their behavior. Finally, our empirical study was conducted in China; therefore, researchers should be cautious about generalizing our findings. We suggest that a larger and multinational sample be utilized to validate these conclusions in other national contexts.

## Conclusion

This study extended the TPB to examine the mediation effect of sharing bike users’ civilized cycling intentions on the relationship between four determinants and their actual civilized cycling behavior and the moderating effect of PPE between users’ civilized cycling intentions and their actual intentional behavior. After controlling for demographic variables including gender, age, education and income, our results show that attitude, PBC, and PN are positively related to both users’ civilized cycling intentions and their actual behavior. SN has a positive impact on users’ civilized cycling intentions only. Furthermore, users’ civilized cycling intentions mediate the relationship of influencing factors (ATT, SN, PBC, and PN) with users’ civilized cycling behavior. Such results indicate that ATT, SN, PBC, and PN motivate users’ civilized cycling intentions, and their actual civilized cycling behavior can occur especially when the intention exists. The results also indicate that PPE has a positive moderating effect on the relationship between users’ civilized cycling intentions and their actual behavior. Policies such as credit-based supervision systems could promote users’ civilized cycling intentions for sharing bikes. When a user perceives a high degree of effectiveness of credit-based supervision policies, the intention-behavior gap would be narrowed by facilitating the realization of the actual intended civilized cycling behavior.

## Data Availability Statement

The datasets generated for this study are available on request to the corresponding author.

## Ethics Statement

The internal Ethics Committee of the Tianjin University approved this study. There were no unethical behaviors in the research process, and all subjects gave written informed consent. For non-adult participants, written informed consent was also obtained from their parents or guardian.

## Author Contributions

LS and XZ developed the original idea for the study. XZ and ZS designed the methodology and analyzed the data. XZ completed the data collecting and drafted the manuscript. LS revised it critically for important content. All authors read and approved the final manuscript.

## Conflict of Interest

The authors declare that the research was conducted in the absence of any commercial or financial relationships that could be construed as a potential conflict of interest.

## Footnotes

1 Ministry of Transport. Encouraging and standardizing the development of bicycle-sharing. Available at: http://xxgk.mot.gov.cn/jigou/ysfws/201708/t20170802_2978814.html.

2 Sharing organizations liking Mobike give each user a 100-point credit score, with points taken in the case of irregularities. If user’s score drops below 80 points, the rental fee for each 30 min will increase from 0.5–1 Yuan to 100 Yuan. Users who accumulate high credit scores for civilized behaviors can exchange them for gifts or apply them to their expenses. In the future, all kinds of dishonest behaviors of both individuals and enterprises will be recorded in the database. https://www.creditchina.gov.cn/home/xinyongdongtaituijian/201709/t20170930_57873.html

## References

[B1] Acheampong R. A. (2017). Towards sustainable urban transportation in Ghana: exploring adults’ intention to adopt cycling to work using theory of planned behaviour and structural equation modelling. *Transp. Dev. Econ.* 3:18 10.1007/s40890-017-0047-8

[B2] Ajzen I. (1991). The theory of planned behavior. *Organ. Behav. Hum. Dec.* 50 179–211. 10.1016/0749-5978(91)90020-T

[B3] Ajzen I. (2006). *Constructing a TPB Questionnaire: Conceptual and Methodological Considerations.* Available at: http://people.umass.edu/aizen/tpb.html (accessed July 19).

[B4] Ajzen I. (2011). The theory of planned behaviour: reactions and reflections. *Psychol. Health* 26 1113–1127. 10.1080/08870446.2011.613995 21929476

[B5] Armitage C. J. Conner M. (2001). Efficacy of the theory of planned behaviour: a meta-analytic review. *Br. J. Soc. Psychol.* 40 471–499. 10.1348/014466601164939 11795063

[B6] Bamberg S. Hunecke M. Blobaum A. (2007). Social context, personal norms and the use of public transportation: two field studies. *J. Environ. Psychol.* 27 190–203. 10.1016/j.jenvp.2007.04.001

[B7] Beijing Municipal Commission of Transport (2018). *Management Order of Bicycle-Sharing Industry.* Available at: http://jtw.beijing.gov.cn/zmhd/zxft/wqhg/201805/t20180523_192981.html (accessed May 23).

[B8] Bernhoft I. M. Carstensen G. (2008). Preferences and behaviour of pedestrians and cyclists by age and gender. *Transport. Res. F-Traf.* 11 83–95. 10.1016/j.trf.2007.08.004

[B9] Bettina H. Adrian B. Roman A. Claude M. (2019). Using a goal theoretical perspective to reduce negative and promote positive spillover after a bike-to-work campaign. *Front. Psychol.* 10:433. 10.3389/fpsyg.2019.00433 30894827 PMC6414797

[B10] Boudrifa H. Bouhafs A. Touil M. Tabtroukia F. (2012). Factors and motives of unsafe behaviors of road users. *Work* 1 4910–4918. 10.3233/WOR-2012-0785-4910 22317479

[B11] Brislin R. W. (1980). “Translation and content analysis of oral and written material,” in *Handbook of Cross-Cultural psycHology: Methodology*, eds Triandis H. Berry J. (London: Allyn & Bacon), 389–444.

[B12] Bruijn G. J. D. Kremers S. P. J. Singh A. Putte B. V. D. Mechelen W. V. (2009). Adult active transportation: adding habit strength to the theory of planned behavior. *Am. J. Prev. Med.* 36 189–194. 10.1016/j.amepre.2008.10.019 19162430

[B13] Buehler R. Pucher J. Gerike R. Götschi T. (2016). Reducing car dependence in the heart of Europe: lessons from Germany, Austria, and Switzerland. *Transport. Rev.* 36 1–25. 10.1080/01441647.2016.1177799

[B14] Cai S. Long X. Li L. Liang H. Wang Q. Ding X. (2019). Determinants of intention and behavior of low carbon commuting through bicycle-sharing in China. *J. Clean. Prod.* 212 602–609. 10.1016/j.jclepro.2018.12.072

[B15] CBS News (2018). *Cities Vow to Crack Down on‘Litter Bikes.* Available at: https://www.cbsnews.com/news/cities-vow-to-crack-down-on-litter-bikes/ (accessed 30 April).

[B16] Chen M. Zhao L. Xia J. (2018). Research on users’ illegal parking behavior of bicycle-sharing based on norm activation theory (Chinese). *Inform. Res.* 8 49–55.

[B17] Cordellieri P. Baralla F. Ferlazzo F. Sgalla R. Piccardi L. Giannini A. M. (2016). Gender effects in young road users on road safety attitudes, behaviors and risk perception. *Front. Psychol.* 7:1412. 10.3389/fpsyg.2016.01412 27729877 PMC5037216

[B18] de Leeuw A. Valois P. Ajzen I. Schmidt P. (2015). Using the theory of planned behavior to identify key beliefs underlying pro-environmental behavior in high-school students: implications for educational interventions. *J. Environ. Psychol.* 42 128–138. 10.1016/j.jenvp.2015.03.005

[B19] Deng J. Sun P. Zhao F. Han X. Yang G. Feng Y. (2016). Analysis of the ecological conservation behavior of farmers in payment for ecosystem service programs in eco-environmentally fragile areas using social psychology models. *Sci. Total Environ.* 550 382–390. 10.1016/j.scitotenv.2016.01.152 26829672

[B20] Du H. Liu D. Sovacool B. K. Wang Y. Rita S. M. Li Y. M. (2018). Who buys new energy vehicles in China? Assessing social-psychological predictors of purchasing awareness, intention, and policy. *Transport. Res. F-Traf.* 58 56–69. 10.1016/j.trf.2018.05.008

[B21] Faghih-Imani A. Hampshire R. Marla L. Eluru N. (2017). An empirical analysis of bike sharing usage and rebalancing: evidence from Barcelona and Seville. *Transport. Res. A-Pol.* 97 177–191. 10.1016/j.tra.2016.12.007

[B22] Federico F. Marín Puchades V. Marco D. A. Gabriele P. Luca P. (2016). Social influence and different types of red-light behaviors among cyclists. *Front. Psychol.* 7:1834. 10.3389/fpsyg.2016.01834 27920747 PMC5118586

[B23] Fishman E. (2016). Bikeshare: a review of recent literature. *Transport. Rev.* 36 92–113. 10.1080/01441647.2015.1033036

[B24] Fishman E. Washington S. Haworth N. (2014). Bike share’s impact on car use: evidence from the United States, Great Britain, and Australia. *Transport. Res. D-TR. E.* 31 13–20. 10.1016/j.trd.2014.05.013

[B25] Fornell C. Larcker D. F. (1981). Evaluating structural equation models with unobservable variables and measurement Error. *J. Mark. Res.* 66 39–50. 10.2307/3150979

[B26] French D. P. Sutton S. Hennings S. Mitchell J. Wareham N. Griffin S. (2005). The importance of affective beliefs and attitudes in the theory of planned behavior: predicting intention to increase physical activity. *J. Appl. Soc. Psychol.* 35 1824–1848. 10.1111/j.1559-1816.2005.tb02197.x

[B27] Guo P. (2017). Bike sharing: collaborative governance in internet technology and public services (Chinese). *J. Public Manag.* 14 1–10. 10.16149/j.cnki.23-1523.2017.03.001

[B28] Hagger M. S. Chatzisarantis N. L. D. Biddle S. J. H. (2011). The influence of autonomous and controlling motives on physical activity intentions within the theory of planned behaviour. *Br. J. Health Psychol.* 7 283–297. 10.1348/135910702760213689 12614501

[B29] Hayes A. F. (2009). Beyond Baron and Kenny: statistical mediation analysis in the new millennium. *Commun. Monogr.* 76 408–420. 10.1080/03637750903310360

[B30] Holland C. Hill R. (2007). The effect of age, gender and driver status on pedestrians’ intentions to cross the road in risky situations. *Accid. Anal. Prev.* 39 224–237. 10.1016/j.aap.2006.07.003 16979132

[B31] Hooper D. Coughlan J. Mullen M. (2008). Structural equation modeling: guidelines for determining model fit. *Electron. J. Bus. Res. Methods.* 6 53–60. 10.1016/j.acap.2015.07.001 26547545

[B32] Huemer A. K. (2018). Motivating and deterring factors for two common traffic-rule violations of cyclists in Germany. *Transport. Res. F-Traf.* 54 223–235. 10.1016/j.trf.2018.02.012

[B33] iiMedia Research (2018). *2018 China Shared Bikes Development Status Research.* Available at: https://www.iimedia.cn/c400/63243.html (accessed July 19).

[B34] Jia L. Liu X. Liu Y. (2018). Impact of different stakeholders of bike-sharing industry on users’ intention of civilized use of bike-sharing. *Sustainability* 10:1437 10.3390/su10051437

[B35] Katharina H. A. (2018). Cycling under the influence of alcohol in Germany. *Transport. Res. F-Traf.* 56 408–419. 10.1016/j.trf.2018.05.013

[B36] Kim J. Choi K. Kim S. Fujii S. (2017). How to promote sustainable public bike system from a psychological perspective? *Int. J. Sustain. Transp.* 11 272–281. 10.1080/15568318.2016.1252450

[B37] Kollmuss A. Agyeman J. (2002). Mind the gap: why do people act environmentally and what are the barriers to pro-environmental behavior? *Environ. Educ. Res.* 8 239–260. 10.1080/13504620220145401

[B38] Li X. Zhang Y. Sun L. Liu Q. (2018). Free-floating bike sharing in Jiangsu: users’ behaviors and influencing factors. *Energies* 11:1664 10.3390/en11071664

[B39] Ma Y. Lan J. Thomas T. Diana M. Zhu D. (2018). Challenges of collaborative governance in the sharing economy: the case of free-floating bike sharing in shanghai. *J. Clean. Prod.* 197 356–365. 10.1016/j.jclepro.2018.06.213

[B40] MacKinnon D. P. Lockwood C. M. Hoffman J. M. West S. G. Sheets V. (2002). A comparison of methods to test mediation and other intervening variable effects. *Psychol. Methods* 7 83–104. 10.1037/1082-989x.7.1.83 11928892 PMC2819363

[B41] MacKinnon D. P. Lockwood C. M. Williams J. (2004). Confidence limits for the indirect effect: distribution of the product and resampling methods. *Multivar. Behav. Res.* 39 99–128. 10.1207/s15327906mbr3901_4 20157642 PMC2821115

[B42] Mátrai T. Tóth J. (2016). Comparative assessment of public bike sharing systems. *Transp. Res. Proc.* 14 2344–2351. 10.1016/j.trpro.2016.05.261

[B43] Mehta P. D. (2015). “Control variables in research,” in *International Encyclopedia of the Social & Behavioral Sciences*, ed. Wright J. (London: Elsevier), 840–843. 10.1016/b978-0-08-097086-8.44013-4

[B44] Ministry of Transport (2017). *Encouraging and Standardizing the Development of Bicycle-Sharing.* Available at: http://xxgk.mot.gov.cn/jigou/ysfws/201708/t20170802_2978814.html (accessed July 19).

[B45] Mullan B. Wong C. Kothe E. (2013). Predicting adolescent breakfast consumption in the UK and Austria using an extending theory of planned behavior. *Appetite* 62 127–132. 10.1016/j.appet.2012.11.021 23219456

[B46] Nikitas A. (2018). Understanding bike-sharing acceptability and expected usage patterns in the context of a small city novel to the concept: a story of ‘Greek Drama’. *Transp. Res. F-Traf.* 56 306–321. 10.1016/j.trf.2018.04.022

[B47] Nordlund A. M. Garvill J. (2003). Effects of values, problem awareness, and personal norm on willingness to reduce personal car use. *J. Environ. Psychol.* 23 339–347. 10.1016/S0272-4944(03)00037-9

[B48] Qu Y. Liu Y. Zhu Q. Liu Y. (2014). Motivating small-displacement car purchasing in china. *Transport. Res. A-Pol.* 67 47–58. 10.1016/j.tra.2014.06.002

[B49] Renger D. Reese G. (2017). From equality-based respect to environmental activism: antecedents and consequences of global identity. *Polit. Psychol.* 38 867–879. 10.1111/pops.12382

[B50] Ross J. Weitzman R. A. (1964). The twenty-seven per cent rule. *Ann. Math. Stat.* 35 214–221. 10.1214/aoms/1177703745 8153001

[B51] Schneider A. Ingram H. (1990). Behavioural assumptions of policy tools. *J. Polit.* 52 510–529. 10.2307/2131904

[B52] Shanghai Municipal Commission of Transport (2017). *A Total of 516,000 Bicycle-Sharing Have Been Cleaned Up in SHANGHAI.* Available at: http://www.shanghai.gov.cn/nw2/nw2314/nw32419/nw42619/nw42622/u21aw1268302.html (accessed November 7).

[B53] Shen Y. Zhang X. Zhao J. (2018). Understanding the usage of dockless bike sharing in Singapore. *Int. J. Sustain. Transp.* 9 1–15. 10.1080/15568318.2018.1429696

[B54] Shi J. G. Si H. Wu G. Su Y. Lan J. (2018). Critical factors to achieve dockless bike-sharing sustainability in China: a stakeholder-oriented network perspective. *Sustainability* 10:2090 10.3390/su10062090

[B55] Si H. Shi J. G. Wu G. Chen J. Zhou X. (2019). Mapping the bike sharing research published from 2010 to 2018: a scientometric review. *J. Clean. Prod.* 213 415–427. 10.1016/j.jclepro.2018.12.157

[B56] State Information Center (2019). *Annual Report on China’s Shared Economic Development (2019).* Available at: http://www.sic.gov.cn/News/568/9906.htm (accessed July 19).

[B57] Sun Y. Mobasheri A. Hu X. Wang W. (2017). Investigating impacts of environmental factors on the cycling behavior of bicycle-sharing users. *Sustainability* 9:1060 10.3390/su9061060

[B58] Tarhini A. Scott M. J. Sharma S. K. Abbasi M. S. (2015). Differences in intention to use educational rss feeds between lebanese and british students: a multi-group analysis based on the technology acceptance model. *Electron. J. e Learn.* 13 14–29.

[B59] Thøgersen J. (2006). Norms for environmentally responsible behaviour: an extended taxonomy. *J. Environ. Psychol.* 26 247–261. 10.1016/j.jenvp.2006.09.004

[B60] Tom S. Q. Brunton J. A. (2017). Implicit processes, self-regulation, and interventions for behavior change. *Front. Psychol.* 8:346. 10.3389/fpsyg.2017.00346 28337164 PMC5340749

[B61] Van Roy J. (2017). *Amsterdam to Ban Free-Floating Bicycle-Sharing.* Available at: https://newmobility.news/2017/08/03/amsterdam-ban-free-floating-bicycle-sharing/ (accessed July 19).

[B62] Wan C. Shen G. Q. (2013). Perceived policy effectiveness and recycling behaviour: the missing link. *Waste Manag.* 33 783–784. 10.1016/j.wasman.2013.02.001 23541259

[B63] Wan C. Shen G. Q. Yu A. (2014). The moderating effect of perceived policy effectiveness on recycling intention. *J. Environ. Psychol.* 37 55–60. 10.1016/j.jenvp.2013.11.006

[B64] Wang C. Zhang J. Yu P. Hu H. (2018). The theory of planned behavior as a model for understanding tourists’ responsible environmental behaviors: the moderating role of environmental interpretations. *J. Clean. Prod.* 194 425–434. 10.1016/j.jclepro.2018.05.171

[B65] Wang Y. Yang J. Liang J. Qiang Y. Fang S. Gao M. (2018). Analysis of the environmental behavior of farmers for non-point source pollution control and management in a water source protection area in china. *Sci. Total Environ.* 633 1126–1135. 10.1016/j.scitotenv.2018.03.273 29758864

[B66] Wheaton B. Muthén B. Alwin D. F. Summers G. F. (1977). Assessing reliability and stability in panel models. *Sociol. Methodol.* 8 84–136. 10.2307/270754

[B67] Williams S. J. Jones J. P. G. Colin C. Gibbons J. M. Karen F. (2012). Training programmes can change behaviour and encourage the cultivation of over-harvested plant species. *PLoS one* 7:e33012. 10.1371/journal.pone.0033012 22431993 PMC3303790

[B68] WRI and Mobike (2018). *How Cycling Changes Cities.* Available at: https://mobike.com/global/public/HowCyclingChangesCitiesMobike.pdf (accessed July 19).

[B69] Wu X. Xiao W. Deng C. Schwebel D. C. Hu G. (2019). Unsafe riding behaviors of shared-bicycle riders in urban China: a retrospective survey. *Accid. Anal. Prev.* 131 1–7. 10.1016/j.aap.2019.06.002 31228635

[B70] Xiao C. Jianqiao L. Weijiong W. Wei Z. (2017). Perceived insider status and feedback reactions: a dual path of feedback motivation attribution. *Front. Psychol.* 8:668. 10.3389/fpsyg.2017.00668 28507527 PMC5410612

[B71] Xu L. Ling M. Lu Y. Shen M. (2017). Understanding household waste separation behaviour: testing the roles of moral, past experience, and perceived policy effectiveness within the theory of planned behaviour. *Sustainability* 9:625 10.3390/su9040625

[B72] Yang L. Zhu D. (2018). Research on influencing factors influencing bicycle-sharing users’ orderly parking intention-based on extended theory of planned behavior(Chinese). *China Popul. Res. Env.* 28 125–133.

[B73] Yao Y. Liu L. Guo Z. Liu Z. Zhou H. (2019). Experimental study on shared bike use behavior under bounded rational theory and credit supervision mechanism. *Sustainability* 11:127 10.3390/su11010127

